# Current Hospital Topics

**Published:** 1909-04-17

**Authors:** 


### HOSPITAL ADMINISTRATION.

### CONSTRUCTION AND ECONOMICS.

### CURRENT HOSPITAL TOPICS.

#### Hospital Tickets.

A good deal of discussion has taken place in Birmingham on the subject of hospital tickets and the difficulty frequently experienced in obtaining these tickets by deserving people who are not known to subscribers. The Hospital Sunday Fund recommended that the various medical charities, before forwarding to their subscribers the tickets to which they were entitled each year, should send out a form to be filled up, whereby the institutions will be empowered to send the whole or any portion of the subscribers' tickets to the City Aid Society as a central distributing office. This recommendation was referred to a sub-committee, who summoned a conference of representatives of medical charities issuing tickets to consider it. Representatives of the General Hospital, the General Dispensary, the Eye Hospital, the Ear and Throat Hospital, the Dental Hospital, the Moseley Hall Convalescent Home, the Orthopædic and Spinal Hospital, and the Sanatorium were present. The Queen's Hospital did not send a representative. The representatives of the General and other hospitals were unwilling to send out a circular in the way suggested. A few of the smaller hospitals supported the suggestion, and in the end it was decided to adjourn the conference to enable the hospitals to consider a draft circular which Mr. Neville Chamberlain, as representing the City Aid Society, had drawn up. It seems to us, as a matter of experience, that the hospital authorities would be wise not to issue the circular suggested, but to leave the City Aid Society, if it thinks well, to address a circular to the subscribers of each of the hospitals, asking them to forward to that society any tickets they may have to spare each year, and to use it as a central agency where poor deserving people who may need hospital relief may obtain tickets on application.

#### The Old Site of the Manchester Royal Infirmary.

For more than thirty years questions relating to this site have given rise to controversy in the city of Manchester. As the *Manchester Guardian* sagely remarks, the infirmary site seems to be destined to perpetual strife. It was the wish of the trustees of the Royal Infirmary that they should sell the site, obtaining for it the best price they could secure. This was objected to at the time on the ground that, being the finest site in the city, it should be utilised for public purposes, and with much reluctance the trustees ultimately agreed to part with their property to the corporation for a very reasonable figure. The trustees were assured that, even if they got a little less than its full market value for their property, still, whatever might be loss to one public object, would be gain to another, and that for all time the infirmary site would be dedicated strictly to public purposes. It appears as if a party in the corporation, led by Sir William Vaudrey, are inclined to disregard the understanding under which the site was purchased from the infirmary trustees, and to sell it to the Royal Exchange proprietors or to others at a high figure. Against any such departure from a clear understanding the *Manchester Guardian* raises a strong protest, which we beg earnestly to support. It would indeed be a reflection upon the good faith and character of the Manchester Corporation, after maintaining through all the years of controversy that the city had certain rights in this site, which placed an obligation upon the trustees to hand it over to the corporation, for the corporation now to ignore their obligations and consent to sell the site to the highest bidder to be used for other than public purposes.

#### The Royal Hospital, Richmond.

Important changes have taken place at the Royal Hospital, Richmond. The retirement of the secretary, Mr. G. C. Rowland, after 37 years' service, on a pension of £100 a year, and the appointment of Mr. Richard Allen to succeed him, is followed by the gratifying announcement of the adoption of the uniform system of accounts, as directed by King Edward's Hospital Fund. This will enable a comparison to be shown in future years between each item in the income and expenditure account, and will lead to the publication of statistical tables which cannot fail to promote the economical administration of the hospital. Colonel R. W. Sparkes, J.P., after 23 years' service, has resigned the office of chairman, and Mr. William Sandover has been appointed his successor. After 18 years' service Miss Foley has retired from the office of matron, but has not been granted a pension for some reason which is not explained in the report, although the committee record their appreciation of her services, and raised a subscription to a purse which was presented to her.

